# Aphorisms on the Hygiene and Nursing of Infants

**Published:** 1857-03

**Authors:** 


*Aphorisms on the Hygiene and Nursing of Infants.* From the last edition of BOUCHUT'S "*Traité Pratique des Maladies des Nouveaux Nés et des Enfans a la Mamelle.*"—Translated by J. C. R., Dayton, Ohio.

The child should be subjected to hygienic regulations from its cradle, in order to sustain its constitution if it is good, in order to ameliorate it if bad.

We must combat, in early infancy, the scrofulous, gouty, and syphilitic dispositions inherited from the parents.

A man with impure blood should never think of perpetuating his race.

A woman who becomes *enceinte*, should renounce those habits, pleasures and fatigues, which may exercise an evil influence upon the health of the fœtus, if she wishes to give birth to a healthy child.

Blood-letting has a good effect upon gestation, but it should not be used unless plethora, local, or general, is present.

Denial of the unreasonable caprices of a pregnant woman cannot have any influence upon the health of the infant.

A woman can and ought to nurse her child, if she is in good health; and if her parents or immediate relations are not scrofulous, consumptive, or cancerous.

There are women of good constitution unable, nevertheless, to nurse, for their milk is small in quantity, badly elaborated, and dries up from the slightest causes.

A woman in whom the mammary secretion is very active previous to her accouchment, is almost always a good nurse.

A mother who nurses, can commence six or eight hours after the birth of the child.

A woman who nurses should not suckle the child oftener than every two hours.

An infant that takes the breast at regular intervals, sucks with more avidity than others, and drains the breast of all the milk it contains—and it is the part last obtained which is the best, as it contains more cream than the first parts of the flow.

Between eleven o'clock at night, and six or seven in the morning, a good nurse need only suckle the child once.

It is dangerous to take, for a hired nurse, a primiparous woman; she is necessarily inexperienced.

A good nurse is from twenty to thirty-five years of age, with brown hair, the gums bright red, the form inclined to *embonpoint*, the breasts well formed, firm, and with bluish veins.

A nurse should not have any mark, recent or ancient, of scrofula or syphilis.

The milk yellowish in the first months after birth, and bluish white afterwards, is an alkaline emulasion formed of water and solid principles dissolved or suspended.

The butter is only suspended in the liquid; the other principles are dissolved.

The milk should be abundant to be profitable.

The first part of the milk drawn from the breasts is serious; the second part is thicker, and it is the last part of the draught which is the richest and the most charged with cream.

The milk (examined by the microscope,) should be filled with globules, numerous, tolerably large, and well formed for small globules, resembling dust, are a sign of its bad elaboration, and of its insufficiency.

Too few, or too many globules, are equally injurious.

The milk varies in its composition according to idiosyncrasy, temperament, constitution, the time elapsed since the accouchment, the time since the last repast, the regimen of the nurse, the action of the genital organs, ete., etc ; but the differences are not so great as to modify the precept : " If the infant thrives, then the milk is good."

The milk is altered in composition by the febrile state, and by acute and chronic diseases

Fever diminishes the quantity of milk, reduces the number of globules, and concentrates its solids in a smaller proportion of water.

The effect is the same, in different degrees, in all acute affections and in some chronic ones.

Pus is sometimes mixed with the milk, in cases of abscess of the breast.

The influence of diseases upon the composition of the milk, is not special and specific, for they all have the same effect which is the same as that of fever.

The milk of a healthy nurse, which is too rich, or too highly charged with solid elements, is indigestible, and causes diarrhœa.

Milk altered, reduced and impoverished, by fever or by disease, also causes diarrhœa.

Milk altered in its composition by fever, or disease, does not always exercise an injurious influence upon the health of the child.

Whatever may be the cause of alteration in the composition of the milk, the result is always the same for the infant—the accidents which arise have always for their seat the digestive canals, and diarrhœa is always the consequence.

Milk which does not present any alteration appreciable to chemical analysis, may yet be altered in its intimate elaboration in such a manner as to make it an injurious aliment.

Spasms, or instantaneous convulsions, result ordinarily from changes caused in the secretion of milk by mental affections, too lively emotions and impressions, agreeable or painful, experienced by the nurse.

Mental impressions dry up, suddenly, the secretion of milk, or modify, seriously, the proportion of its solid elements.

The happiness which a woman feels in fulfilling her duties of nurse, is the cause of the internal sensation, at the moment she is going to nurse the child, known as the *draught*.

The premature return of menstruation in a nurse, modifies, slightly, the chemical composition of the milk, and injures its elaboration; but if the infant does not appear to suffer, which often happens, the nurse may be retained.

A nurse should abstain from sexual intercourse, if she experiences great excitement.

A nurse should likewise abstain through fear of pregnancy, which modifies the milk in quantity and quality, so as to render it injurious to the child.

A change of nurses has no injurious effects, when necessary to replace a poor one by a better.

The nurse should be changed as often as may be necessary.

Suckling, by mother or nurse, may give place to artificial feeding.

Feeding by the nursing-bottle is far inferior to suckling—although when well carried on it sometimes yields highly satisfactory results.

Artificial food should be administered during the earliest periods of life, means of nursing bottle, filled with tepid milk, diluted with barley-water, or oat-meal gruel ; afterwards with milk alone.

An infant needs nothing more than milk, during the first months of life. At the age of six months it may commence to take light soups.

Greasy articles of food should not be given until after the first year.

The time of weaning should be fixed between the twelfth and twentieth month.

One of the periods of repose in the progress of dentition, should be chosen for weaning—that which comes after the appearance of the first twelve, or of the first sixteen teeth.

Weaning should be commenced by keeping the child from the breast during the night.

After some weeks' separation from the mother at night, the child should be denied the breast in the day time also, and it thus arrives at an independent existence.

Infants and children should be carried into the sunlight and open air in all kinds of weather.

Clothes which fit the body, without constriction, are preferable, in all weathers, to loose ones, which expose different portions of the skin to the cold.

Infants should be washed in tepid water, every day, and as they become habituated to it, in water nearly cold.—*Western Lancet.*

